# Supplementary material for: The mouse suprachiasmatic nucleus encodes irradiance via a diverse population of neurons monotonically tuned to different ranges of intensity
Source: J Physiol. 2023 Oct 1;601(21):4737–49. doi: 10.1113/JP285000 (PMC10953322; doi:10.1113/JP285000)
Supplement: Supplementary file 3 — Supporting Information [file TJP-601-4737-s002.docx]

**Supporting Information**

**Title:** The mouse suprachiasmatic nucleus encodes irradiance via a diverse population of neurons monotonically tuned to different ranges of intensity.

Patrycja Orlowska-Feuer^1*^, Beatriz Bano-Otalora^2^, Jessica Rodgers^1^, Franck P. Martial^1^, Riccardo Storchi^1^, Robert James Lucas^1*^

^1^ Division of Neuroscience, School of Biological Sciences, Faculty of Biology, Medicine and Health, University of Manchester, Manchester, UK

^2^ Centre for Biological Timing, Faculty of Biology, Medicine and Health, University of Manchester, Manchester M13 9PT, UK

^*^ Corresponding authors [patrycjaanna.orlowska-feuer@manchester.ac.uk](mailto:patrycjaanna.orlowska-feuer@manchester.ac.uk), [robert.lucas@manchester.ac.uk](mailto:robert.lucas@manchester.ac.uk)

**Supporting Information for Figure 3A-C**


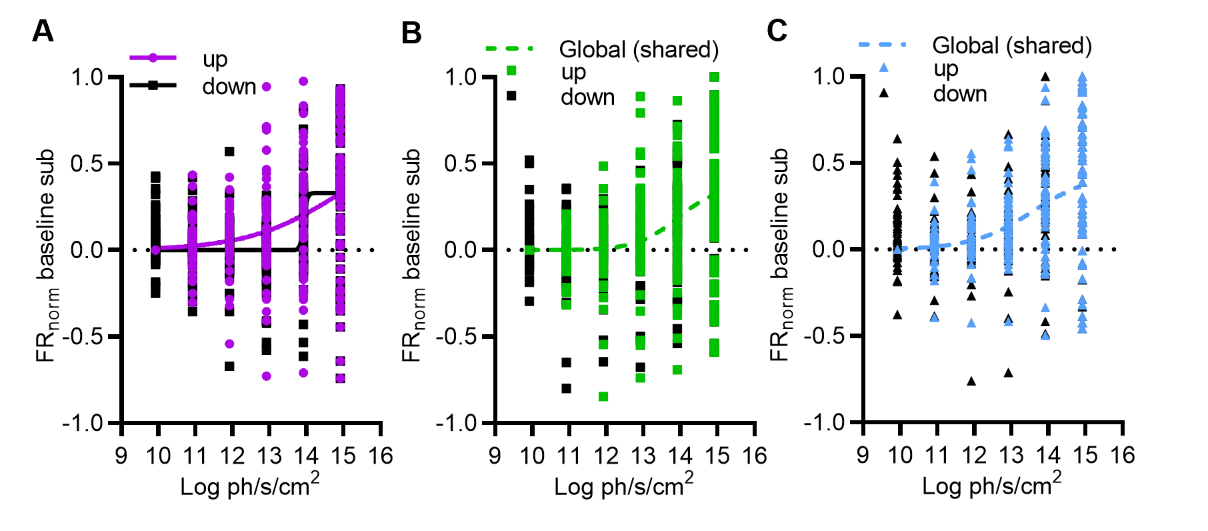


**Irradiance response relationships for the population of SCN light responsive units**. A, B, C) Baseline subtracted, normalised firing rate, averaged across repeats of the staircase for individual units (n=57), as a function of irradiance for ascending (up) and descending (down) phases (coloured vs black points respectively) for steady (A), chirp (B) and WN (C) phases of the step. Curves show sigmoidal function fit to combined ascending and descending phases for chirp and WN, but separately for steady light (F-test, steady: p=0.0256, chirp: p=0.3097, WN: p=0.3843).
